# Oral, Vaginal and Anal Sexual Practices among Heterosexual Males and Females Attending a Sexual Health Clinic: A Cross-Sectional Survey in Melbourne, Australia

**DOI:** 10.3390/ijerph182312668

**Published:** 2021-12-01

**Authors:** Tiffany R. Phillips, Heidi Constantinou, Christopher K. Fairley, Catriona S. Bradshaw, Kate Maddaford, Marcus Y. Chen, Jane S. Hocking, Eric P. F. Chow

**Affiliations:** 1 Melbourne Sexual Health Centre, Alfred Health, Melbourne, VIC 3053, Australia; heidiconstantinou@gmail.com (H.C.); cfairley@mshc.org.au (C.K.F.); cbradshaw@mshc.org.au (C.S.B.); kmaddaford@mshc.org.au (K.M.); mchen@mshc.org.au (M.Y.C.); eric.chow@monash.edu (E.P.F.C.); 2 Central Clinical School, Monash University, Melbourne, VIC 3800, Australia; 3 Centre for Epidemiology and Biostatistics, Melbourne School of Population and Global Health, The University of Melbourne, Parkville, VIC 3010, Australia; j.hocking@unimelb.edu.au

**Keywords:** condoms, sexual behaviour, intercourse, survey

## Abstract

Sex practices among heterosexuals are not well studied. We aimed to explore sexual practices among heterosexuals attending a sexual health clinic. This cross-sectional survey was conducted at Melbourne Sexual Health Centre between March and April 2019. Data were collected on kissing, oral sex (fellatio or cunnilingus), vaginal sex, anal sex and rimming in the previous 3 months. Univariable and multivariable logistic regression analyses were performed to examine the associations between engaging in anal sex and other sex practices. There were 709 participants (333 men; 376 women) who were eligible and completed the survey (response rate was 24.6%). In the past 3 months, most participants had had vaginal sex (*n* = 677; 95.5%), with a mean of 3.0 (standard deviation (SD): 3.9) vaginal sex partners, and half reported engaging in condomless vaginal sex in the past 3 months (*n* = 358; 50.1%). A total of 135 (19.0%) participants had had anal sex, with a mean of 1.3 (SD: 1.0) anal sex partners, with 63.5% (*n* = 94) engaging in any condomless anal sex in the past 3 months. Most participants (*n* = 637, 89.8%) had received oral sex in the past 3 months; this proportion did not differ by age group or gender. Women (*n* = 351, 93.4%) were more likely to perform oral sex than men (*n* = 275; 82.6% men) (*p* < 0.001) and to have received rimming (26.6% women vs. 12.6% men; *p* < 0.001). Men were more likely to have performed rimming (25.5% men vs. 9.3% women; *p* < 0.001). After adjusting for age, number of partners and sexual practice, anal sex was associated with being ≥35 years (adjusted odds ratio (aOR): 2.3; 95% CI: 1.2–4.2), receiving rimming (aOR: 3.8; 95% CI: 2.4–6.0) and performing rimming (aOR: 2.8; 95% CI: 1.8–4.6). Rimming and anal sex are practiced by one-fifth or more of heterosexuals. Older heterosexuals were more likely to engage in anal sex and to perform rimming. Future research should consider the benefits of testing extragenital sites where appropriate.

## 1. Introduction

Sexually transmitted infections (STIs), such as gonorrhoea and syphilis, have been on the rise in heterosexuals in the last decade in Australia [1,2,3] and other developed countries [4,5]. There was also a 10% increase in HIV notifications for heterosexuals in Australia between 2013 and 2017 [6]. Despite these rises, much of the behavioural surveillance data on sexual practices focuses on men who have sex with men (MSM). The most recent comprehensive report of heterosexual sex practices in Australia, to our knowledge, was the Second Australian Study of Health and Relationships (ASHR2), a representative survey of the general population in 2011–2012 [7], which was almost 10 years ago.

Data on recent anal sex practices among heterosexuals are particularly lacking, and those studies that examine anal sex practice often report lifetime anal sex practice rather than recent anal sex experiences. Previous estimates of anal sex among heterosexuals vary, with one US study from 2013 finding around a third of heterosexuals had engaged in anal sex in the previous 12 months [8] and another from 2015 finding up to 33% of women and 38% of men had ever engaged in anal sex in their lifetime [8,9]. In contrast, the ASHR2 study in Australia found 25.3% of men and 19.3% of women had ever engaged in anal sex in their lifetime [7].

Recent studies have indicated a rise in STIs, such gonorrhoea and chlamydia, at extragenital sites (i.e., the anus and oropharynx) in both MSM [10,11,12] and heterosexuals [13,14,15], and there is an ongoing debate about whether women reporting anal sex should also be tested for rectal STIs. In addition to the scarcity of data on recent anal sex practice in heterosexuals, there are also limited data on kissing in this population, a practice that has been implicated in the transmission of oropharyngeal gonorrhoea [10,16,17]. Therefore, the aim of this study was to provide a detailed understanding of the recent sexual practices among sexually active heterosexuals, particularly in terms of kissing, oral sex, rimming, anal sex, partner number and age patterns, and to investigate factors associated with anal sex in order to inform STI screening practices in this population.

## 2. Materials and Methods

### 2.1. Study Setting and Procedure

This cross-sectional survey was conducted at the Melbourne Sexual Health Centre (MSHC). MSHC is the largest public sexual health service in Victoria in Australia, providing over 50,000 consultations a year. The ‘Annual Sexual Practices and Activities (ASAP)’ survey was disseminated electronically after clients completed computer-assisted self-interview (CASI), which new clients and those who have not been seen at MSHC in over three months are invited to complete [18]. Between March and April 2019, clients completing CASI were shown an invitation to participate in the ASAP survey if they were aged 16 years or older. Clients were asked to select ‘Yes’ or ‘No’ to consent to participate in the ASAP survey on CASI, and only those participants who selected ‘Yes’ on the invitation were shown the ASAP survey. Based on clients’ demographics and self-reported sexual practices on CASI in the previous 12 months, they were shown a tailored version of ASAP (e.g., ASAP questions for women or ASAP questions for men who did not report having sex with men). The ASAP surveys which were shown to men and women asked additional questions on sexual practices, including the number of same- and opposite-sex partners they kissed but did not have sex with, number of opposite-sex partners they had sex with but did not kiss and number of opposite-sex partners they both kissed and had sex with. The ASAP survey also asked about specific oral and anal sex practices, which CASI does not ask, and about the number of opposite-sex partners in the previous three months with which they had received and performed oral sex (fellatio or cunnilingus), rimming and engaged in vaginal sex, condomless vaginal sex, anal sex and condomless anal sex.

In this analysis, we defined men and women as “heterosexual” if they reported no same-sex sexual partners or same-sex activity (excluding kissing) in the previous 12 months. Self-reported sexual orientation was not collected, thus the term heterosexual throughout this manuscript is based on behaviour and not self-reported sexual identity. Ethics approval was obtained from the Alfred Hospital Ethics Committee, Melbourne, Australia (Project Number 571/17).

### 2.2. Data Analyses

Descriptive statistics were calculated to describe the demographic profile of the participants. The mean age and partner number were calculated, as well as the proportion of men and women who engaged in each sexual practice. Participants were categorised into three age groups (≤24, 25–34 and ≥35 years or older) for analysis, as per previous studies [19,20]. The Mann–Whitney *U* test was used to compare the mean age and number of opposite-sex partners in the previous three months between participants who consented and completed the ASAP survey and those who declined to participate. The χ^2^ test was used to compare categorical variables, and the Mann–Whitney *U* test was used to compare continuous variables between men and women. Cuzick’s nonparametric test for trend was used to compare sex practices between age groups. The proportion engaging in each sexual practice was reported, and the 95% confidence intervals (CI) were calculated using exact binomial methods. Univariable logistic regression was performed to examine factors associated with engaging in anal sex in the previous three months. Variables with *p* < 0.1 in the univariable analysis were included in the multivariable logistic regression.

All statistical analyses were performed using Stata (version 14; College Station, TX, USA).

## 3. Results

There were 2961 heterosexual men and women who were invited to participate in ASAP by CASI, of whom 728 (24.6%) provided consent and completed the survey (*n* = 345, 47.4% men and *n* = 383, 52.6% women). There was no significant difference in mean age between the 728 participants who consented and answered the survey and those who declined (28.6 years vs. 29.1 years; *p* = 0.163). There was no significant difference in the mean number of opposite-sex partners in the previous three months between those who participated and those who did not (2.5 for those who consented vs. 2.4 for those who did not; *p* = 0.104). We excluded 19 (2.6%) of the 728 participants because they had no sexual partners (including kissing-only partners) in the previous three months, leaving 709 participants for analysis.

### 3.1. Sexual Practices

Among the 709 participants included in the analysis, 333 (47.0%) were men, and 376 (53.0%) were women. Men were older than women (*p* < 0.001) (Table 1); the mean age for men was 30.7 (standard deviation (SD): 9.3) and for women was 26.7 (SD: 6.5). Almost all participants (703; 99.2%) had had kissing partners in the previous three months (with and without sex), and this did not differ between the sexes (*p* = 0.332). However, women were more likely than men to have reported same-sex kissing partners (13.3% (50/376) women versus 3.9% (13/333) men; *p* < 0.001). Additionally, a higher proportion of those aged 18–24 years had same-sex kissing partners than those aged 35 and older (12.4% (30/242) of 18–24 years vs. 2.6% (3/115) ≥35 years; *p* = 0.009). There was no significant difference in the proportion who had same-sex kissing partners between those aged 18–24 and those aged 25–34 (8.5% (30/353); *p* = 0.124). There was also no significant difference in the number of kissing partners men and women had in the previous three months (Table 2).

**Figure 1:**
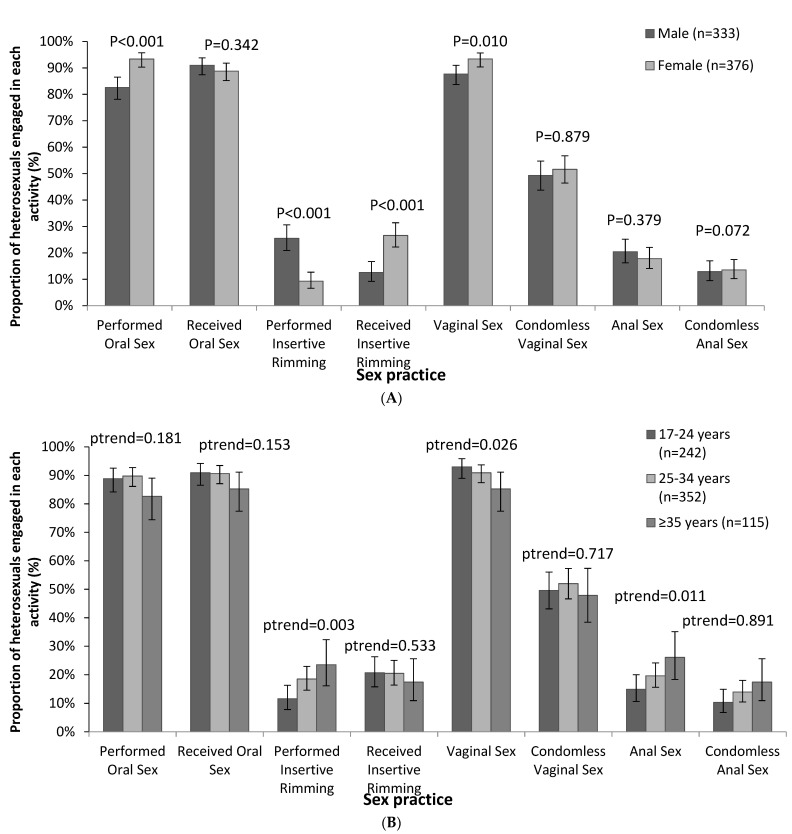
Proportion of heterosexuals who engaged in oral sex, rimming, vaginal and anal sex by (**A**) sex and (**B**) age groups.

Most participants had received oral sex in the previous three months (89.8% (637/709)), and there was no difference in the proportion who received oral sex between the sexes (*p* = 0.342; Supplementary Table S1). Of the men who received oral sex (91.0% (303/333)), there were 261 (86.1%) who received condomless oral sex. Similarly, most participants had performed oral sex (88.3% (626/709)); however, women were more likely than men to have performed oral sex (93.4% (351/376) women versus 82.6% (275/333) men; *p* < 0.001; Figure 1A and Supplementary Table S1). Participants had a mean of 2.6 (standard deviation (SD): 3.8) partners with whom they performed and 2.5 (SD: 3.1) with whom they received oral sex (Table 2), and this did not change between men and women.

There were 142 (20.0%) participants who reported receiving rimming in the previous three months, and women were more likely to receive rimming (26.6% (100/376) women versus 12.6% of men (42/333 *p* < 0.001; Figure 1A and Supplementary Table S1)). Conversely, men were more likely to perform rimming than women (*n* = 85; 25.5% (85/333) of men versus 9.3% (35/376) of women; *p* < 0.001). The mean number of partners with whom participants performed rimming (among those who performed rimming) was 1.7 (SD: 1.9) and among those who received rimming was 1.5 (SD: 1.1), and neither differed between men and women (Table 2).

Most participants reported vaginal sex in the previous three months (95.5% (677/709)), and the mean number of vaginal sex partners among those who had vaginal sex was 3.0 (SD: 3.9; Table 2). Of these, half reported having any condomless vaginal sex in the previous 3 months (50.1% (358/677), and there was no difference in the proportion of men vs. women who engaged in condomless vaginal sex (Supplementary Table S1). There were 135 (19.0%) participants who had anal sex in the previous three months (50.3% (68/135) men and 49.6% (67/135) women; *p* = 0.379), 94 of whom (63.5%) had condomless anal sex, and this did not differ by sex (*p* = 0.072). The mean number of anal sex partners for those who engaged in anal sex in the previous three months was 1.3 (SD: 1.1; Table 2). Among those who engaged in anal sex, there was no significant difference in vaginal or anal sex partner numbers between men and women (*p* = 0.796 for vaginal sex, and *p* = 0.765 for anal sex). Among all 709 participants, the mean vaginal sex partner number was higher than the anal sex partner number (2.9 vs. 0.3; *p* < 0.001).

### 3.2. Associations with Engaging in Anal Sex

After adjusting for age, the number of vaginal sex partners, performing and receiving oral sex and rimming, and condomless vaginal sex in the previous three months, those who received rimming had the highest odds of engaging in anal sex (adjusted odds ratio (aOR): 3.8; 95% CI: 2.4–6.0; Table 3). Similarly, those who performed rimming had 2.8 times higher odds of engaging in anal sex (95% CI: 1.8–4.6). The only other significant association with engaging in anal sex was being aged 35 years or older (aOR: 2.3; 95% CI: 1.2–4.2).

There was a high degree of overlap between engaging in anal sex and receptive and insertive rimming. Almost half (47.4% (64/135)) of those who engaged in anal sex had received insertive rimming compared to only 13.6% (78/574) of those who did not engage in anal sex (*p* < 0.001). Similarly, 40.0% (54/135) of participants who engaged in anal sex also performed rimming versus 11.5% (66/574) who did not engage in anal sex (*p* < 0.001). The correlation between anal sex and insertive and receptive rimming remained for both men and women separately (Supplemental Table S2).

Of the 67 women who had engaged in anal sex in the previous three months, there were 7 (10.4%) who had had an anal swab taken on the day, one of whom was NAAT positive for gonorrhoea.

### 3.3. Age Pattern of Sexual Practices

Younger participants aged 18–24 years were less likely to perform insertive rimming than those aged 35 years and older (11.6% (26/242) of those 18–24 years vs. 23.5% (27/115) of those aged ≥35 years; ptrend = 0.011; Figure 1B and Supplemental Table S3). Similarly, those aged ≥35 years were more likely than those 18–24 years to engage in anal sex (14.9% (36/242) of those 18–24 years vs. 26.1% (30/115) of those aged ≥35 years; ptrend = 0.039). Alternatively, the proportion engaging in vaginal sex was lower among those aged ≥35 years compared to 18–24 years (93.0% (225/242) of those aged 18–24 years vs. 85.2% (98/115) of those aged ≥35 years; ptrend = 0.026). There were no other significant age patterns reported for the other sex practices (oral sex, receptive rimming and condomless vaginal and anal sex).

## 4. Discussion

Our study provides detailed data on sexual practices of 709 sexually active heterosexuals attending a sexual health clinic in Melbourne, Australia. We found that one-fifth of participants had engaged in anal sex and receptive rimming in the previous three months, and a majority of those who engaged in anal sex had condomless anal sex. Engaging in both receptive and insertive rimming was highly correlated with engaging in anal sex. Additionally, our findings indicate an age pattern with anal sexual practice, with those aged 35 years and older more likely than their younger counterparts to perform rimming and engage in anal sex. We also showed variations in sexual behaviour by sex, with a higher proportion of women performing oral sex and having same-sex kissing partners than men. These findings highlight the importance of clinicians asking heterosexual attendees about anal and oral sex activities as part of routine sexual health consultations and potentially including screening of the anal site where indicated. Further research is necessary to investigate whether it would be beneficial to conduct anal STI screening for women who engage in anal sex as gonorrhoea infection is rarely isolated to the anus in women and chlamydia infection has been found in the anus in absence of anal sex due to autoinoculation [21,22,23,24]. Previous studies of anal sex and rimming have primarily focused on men who have sex with men. Our study is one of the first to show that among sexually active heterosexuals, engaging in anal sex and rimming are not uncommon and frequently occur together.

It is difficult to compare the proportion of heterosexuals who engaged in anal sex in the previous 3 months reported in our study to previous studies given the different time frames for reporting that were used. Several studies report anal sex at the last sexual encounter, where the proportion would be expected to be lower for heterosexuals than in the previous three months [25]. However, our findings (for three months) are of a similar magnitude to the previous findings from the Second Australian Study of Health and Relationships (ASHR2) survey from 2011 to 2012, which showed 25.3% of men and 19.3% of women had ever in their lifetime engaged in anal sex, suggesting that the ASHR2 may have under ascertained this practice [7]. A cross-sectional study in the US from the National Survey of Sexual Health and behaviour (2010) showed that between 10.3% and 14.4% of women aged 18 to 39 [26] and between 5.9 % and 15.9% of men aged 18 through 39 engaged in anal sex in the previous three months [27]. In contrast, the proportion of our participants (men and women) aged 17 to 35 who engaged in anal sex was slightly higher, between 14.9% and 19.6%; however, it should be noted that the US study was population based and included individuals with same-sex sexual behaviours. Nevertheless, it could be that more heterosexuals are engaging in anal sex since 2010 or that the proportion is higher among individuals attending a sexual health clinic compared to the general population.

Our findings that older heterosexuals were more likely than their younger counterparts to engage in insertive rimming and anal sex in the previous three months may be because older heterosexuals may have more varied sexual experiences. A comparison of national surveys among young people in the UK disseminated in 1990, 1999 and 2012 showed an increase in the proportion who had ever engaged in anal sex between those aged 16 to 24 years [28]. While our study does not have sufficient power to compare anal sex practice between those aged 16 and those aged 24 (our survey was available to those aged 16 and older but no one aged 16 years was included in the final analysis), our findings showed a similar age pattern with a higher proportion of older individuals engaging in anal sex and insertive rimming compared to younger heterosexuals.

Our findings that men were less likely to receive insertive rimming than women may not be surprising given previously described social stigma among heterosexual men that receiving sexual anal pleasure may indicate they have homosexual tendencies, a concept called homohysteria [29]. A recent qualitative study in the United States of mostly young heterosexual men aged 19 to 33 years suggested there is continuing sexual stigma surrounding anal penetration, despite most participants indicating a level of acceptance of anal stimulation in theory [30].

No Australian study to our knowledge has reported the proportion of women who are not sex workers who have kissing-only partners. However, a previous cross-sectional survey from Melbourne, Australia, among 2351 heterosexual men in 2016–2017 showed 76.1% of men had kissing-only partners in the previous three months [31], a slightly higher proportion than our study (65.5% of men had kissing-only partners). Our finding that women were significantly more likely than men to have same-sex kissing partners may reflect the stigmatisation of same-sex kissing among heterosexual men [32]. A mixed-methods study of heterosexual college students in the United States in 2018 found that of 517 heterosexual men, 10% reported ever kissing another man on the lips [32]. This is lower than the estimate in our study showing only 3.9% of men kissed another man, and this is likely because we specifically asked participants to report ‘tongue kissing’, which may be more sexual than simply kissing on the lips, as the US study indicated most respondents who had same-sex kissing on the mouth indicated it was not romantic. Our sample size did not have sufficient power to make meaningful inferences about age pattern for same-sex kissing in heterosexual men; future research could consider exploring this further given the growing acceptance of same-sex activity [33]. It may be that younger men are more inclined to have same-sex kissing partners. This could have implications for STI transmission given studies have proposed kissing may be a risk factor for oropharyngeal gonorrhoea transmission [16].

Participants in our study showed a high rate of condomless vaginal and anal sex. Combined with the high proportion of heterosexuals who engage in rimming, our study indicates many heterosexuals are engaging in sexual practices that may put them at an increased risk of STIs. Routine anal STI testing is not currently recommended at our clinic or in Australia generally, and thus we were not able to examine whether this practice places heterosexuals at risk of anal STIs. However, there were eight women in our study who had an anal swab on the day, indicating they are sometimes performed at clinician discretion.

Our data were collected before the start of the COVID-19 pandemic (the first Australian cases of COVID-19 were identified in January 2020), and therefore, we are unable to comment on any changes in sexual practices among heterosexuals due to the pandemic. However, previous studies have shown that there was a reduction in self-reported sexual activities and asymptomatic HIV/STI screening in heterosexuals during the COVID-19 pandemic in Australia [34,35,36]. Ongoing behavioural surveillance is warranted among heterosexuals in order to understand STI and HIV risk and to inform best clinical practice.

There are several limitations to this study. First, our study was conducted at one sexual health centre among sexually active individuals, and thus, our findings may not be generalisable to all heterosexuals throughout Australia. Secondly, our participation rate was low for this survey (25.8%), perhaps because the survey was placed after CASI survey fatigue may have played a role. While we reported no significant differences in age and number of sex partners between those who consented and those who did not, it is not possible to know whether participants varied in sexual practices compared to those who did not participate. Thirdly, we did not ask participants to clarify which practices were performed with casual versus regular partners. Previous studies have shown men are more likely to perform rimming with regular partners than casual partners [37], which may have implications for STI risk in this population. Further research is needed to clarify the heterosexual sex practices among casual and multiple partners. Additionally, we asked about sexual practices with partners in the previous 3 months only. While this may reduce recall bias and provide more accurate information, it may not reflect yearly practice. Fourthly, we did not stratify our analysis by ethnicity, and previous studies have shown variations in kissing practices by ethnicity [31]. Furthermore, a high proportion of our participants were born overseas (63%). There were no differences in the proportion born overseas among our participants versus those who declined to consent to the study (64%, *p* = 0.051; data not shown); however, it is unclear why the proportion is so high. It could be that there are more individuals from overseas who are not eligible for Medicare (government-funded healthcare in Australia) attending the clinic, as it is free of cost. Future research could investigate this further in heterosexuals. Finally, neither CASI nor the ASAP survey asks participants to disclose their sexual orientation, and we have defined individuals as ‘heterosexuals’ if they did not have same-sex activity (aside from kissing) in the previous 12 months. It is possible there were individuals in our sample that do not identify as heterosexual.

## 5. Conclusions

Nearly one in five sexually active heterosexuals engaged in anal sex and insertive rimming in the previous 12 months, and there is a significant association between practicing rimming and engaging in anal sex. Almost all heterosexuals reported tongue kissing, and a majority reported receiving and performing oral sex, though the proportion of women performing oral sex was higher than men. In contrast, men were more likely to perform rimming than women. There is a significant age pattern among sexually active heterosexuals for practicing anal sex and insertive rimming, with those 35 years and older more likely to engage in these activities than those aged 16–24 years. Future studies should investigate whether anal STI screening should be performed for heterosexuals engaging in anal sex practices, nevertheless our findings indicate that anal sex practices should be investigated in routine sexual health history taking for heterosexuals.

## Figures and Tables

**Table 1:** Demographics and sexual practice of heterosexual men and women (*n* = 709).

	No. of Individuals (%)	No. of Men (%)	No. of Women (%)	*p*-Value ^a^
Age (year)				<0.001 *
17–24	242 (34.1)	82 (24.6)	160 (42.5)	
25–34	352 (49.7)	168 (50.5)	184 (48.9)	
≥35	115 (16.2)	83 (24.9)	32 (8.5)	
Country of birth				0.002 *
Australia	250 (35.3)	140 (42.0)	110 (29.3)	
Overseas	445 (62.8)	188 (56.5)	257 (68.4)	
Unknown/missing	14 (2.0)	5 (1.5)	9 (2.4)	
Injecting drug use ^b^				0.433
No	696 (98.2)	329 (98.8)	367 (97.6)	
Yes	5 (0.7)	2 (0.6)	3 (0.8)	
Declined to answer	8 (1.1)	2 (0.6)	6 (1.6)	
Had a regular sex partner ^b^				0.003 *
No	406 (57.3)	171 (51.4)	235 (62.5)	
Yes	303 (42.7)	162 (48.7)	141 (37.5)	
Had a casual sex partner ^b^				<0.001 *
No	170 (24.0)	100 (30.0)	70 (18.6)	
Yes	539 (76.0)	233 (70.0)	306 (81.4)	
Had kissing-only partners ^b^				0.717
No	240 (33.9)	115 (34.5)	125 (33.2)	
Yes	469 (66.2)	218 (65.5)	251 (66.8)	
Any tongue kissing ^b^				0.332
No	6 (0.9)	4 (1.2)	2 (0.5)	
Yes	703 (99.2)	329 (98.8)	374 (99.5)	
Kissed same-sex partner ^b^				<0.001 *
No	646 (91.1)	320 (96.1)	326 (86.7)	
Yes	63 (8.9%)	13 (3.9)	50 (13.3)	
Had sex-only partners ^b^				0.215
No	468 (66.0)	212 (63.7)	256 (68.1)	
Yes	241 (34.0)	121 (36.3)	120 (31.9)	

^a^ Using χ^2^ test for significance; ^b^ Participants were asked to report sex partners, injecting drug use, and sex practices that occurred in the previous three months. * Indicates significance (*p* < 0.05).

**Table 2:** Comparison of mean partner number for each sex practice between male and female heterosexuals.

	Mean Total Partner Number (SD) ^a^	Mean Number of Partners for Males (SD) ^a^	Mean Number of Partners for Females (SD) ^a^	*p*-Value ^b^
Kissing only	5.5 (14.9)	5.6 (16.4)	5.5 (13.6)	0.484
Sex only	2.4 (2.4)	2.4 (2.4)	2.4 (2.4)	0.569
Kissing and any sex	3.6 (8.8)	3.9 (11.9)	3.4 (4.6)	0.215
Performed oral sex ^c^	2.6 (3.8)	2.3 (2.3)	2.8 (4.6)	0.947
Receptive oral sex ^d^	2.5 (3.1)	2.7 (2.6)	2.4 (2.0)	0.097
Performed rimming	1.7 (1.9)	1.8 (2.2)	1.3 (0.6)	0.118
Receptive rimming	1.5 (1.1)	1.6 (1.5)	1.5 (0.9)	0.243
Vaginal sex	3.0 (3.9)	2.9 (2.7)	3.1 (4.7)	0.796
Vaginal sex with condom	2.1 (2.8)	1.9 (1.8)	2.2 (3.4)	0.873
Anal sex	1.3 (1.0)	1.2 (0.5)	1.3 (1.4)	0.765
Anal sex with condom	1.2 (0.5)	1.2 (0.5)	1.2 (0.5)	0.683

^a^ Mean partner numbers for each activity are calculated excluding those who did not engage in that activity; ^b^ Calculated using *t*-test; ^c^ Number of partners participant performed oral sex on; ^d^ Number of partners that performed oral sex on the participant.

**Table 3:** Factors associated with engaging in anal sex in the previous three months among heterosexual men and women (*n* = 709).

	No. of Individuals (%)	OR (95 %CI)	*p*-Value	Adjusted OR (95% CI)	*p*-Value
Sex					
Male	333 (47.0)	1 (ref)			
Female	376 (53.3)	0.8 (0.6–1.2)	0.379		
Age (year)					
17–24	242 (34.1)	1 (ref)		1 (ref)	
25–34	352 (49.7)	1.4 (0.9–2.2)	0.139	1.4 (0.8–2.2)	0.207
≥35	115 (16.2)	2.0 (1.2–3.5)	0.012	2.3 (1.2–4.2)	0.009 *
Country of birth					
Australia	250 (35.3)	1 (ref)			
Overseas	445 (62.8)	1.0 (0.7–1.5)	0.923		
Unknown/missing	14 (2.0)	1.2 (0.3–4.4)	0.807		
Number of vaginal sex partners					
≤2	437 (61.6)	1 (ref)		1 (ref)	
>2	272 (38.4)	1.8 (1.3–2.7)	0.002	1.4 (0.9–2.1)	0.174
Performed oral sex ^a^					
No	83 (11.7)	1 (ref)		1 (ref)	
Yes	629 (88.3)	3.3 (1.4–7.8)	0.006	1.5 (0.6–3.0)	0.358
Received oral sex ^b^					
No	72 (10.2)	1 (ref)		1 (ref)	
Yes	637 (89.8)	3.4 (1.4–8.7)	0.009	1.8 (0.6–4.8)	0.267
Performed insertive rimming ^c^					
No	589 (83.1)	1 (ref)		1 (ref)	
Yes	120 (16.9)	5.1 (3.3–7.9)	<0.001	2.8 (1.8–4.6)	<0.001 *
Received rimming ^d^					
No	567 (80.0)	1 (ref)		1 (ref)	
Yes	142 (20.0)	5.7 (3.8–8.7)	<0.001	3.8 (2.4–6.0)	<0.001 *
Condomless vaginal sex					
No	319 (45.0)	1 (ref)		1 (ref)	
Yes	358 (50.5)	1.9 (1.3–2.8)	0.001	1.5 (0.9–2.3)	0.091
N/A No vaginal sex	32 (4.5)	0.4 (0.1–1.7)	0.215	0.8 (0.2–4.1)	0.802

^a^ Oral sex was defined as performing fellatio for females and performing cunnilingus for males; ^b^ Receiving cunnilingus for women and fellatio for males; ^c^ Insertive rimming means participant’s tongue in and around sex partner’s anus; ^d^ Receiving rimming means partner’s tongue in and around participant’s anus. * Indicates significance (*p* < 0.05).

## Data Availability

All data relevant to the study are included in the article or uploaded as Supplementary Information.

## Supplementary Materials

The following are available online at https://www.mdpi.com/article/10.3390/ijerph182312668/s1, Figure S1: Proportion of (A) heterosexual men and (B) heterosexual women who engaged in condomless vaginal sex, anal sex, insertive rimming and receptive rimming by age groups. Table S1: Sexual practice of heterosexual men and women (*n* = 709) in the previous three months. Table S2: Cross-tabulation of anal sex and insertive and receptive rimming among 709 heterosexual men (*n* = 333) and women (*n* = 376). Table S3: Sexual practice of heterosexual men and women (*n* = 709) by age group in the previous three months.
