# Supplementary material for: Oral, Vaginal and Anal Sexual Practices among Heterosexual Males and Females Attending a Sexual Health Clinic: A Cross-Sectional Survey in Melbourne, Australia
Source: Int J Environ Res Public Health. 2021 Dec 1;18(23):12668. doi: 10.3390/ijerph182312668 (PMC8657198; doi:10.3390/ijerph182312668)
Supplement: Supplementary file 1 [file ijerph-18-12668-s001.zip › ijerph-1458888-supplementary.pdf]

Supplementary:

Supp. figure S1: Proportion of (A) heterosexual men and (B) heterosexual women who engaged in condomless vaginal sex, anal sex, insertive rimming and receptive rimming by age groups.

A.

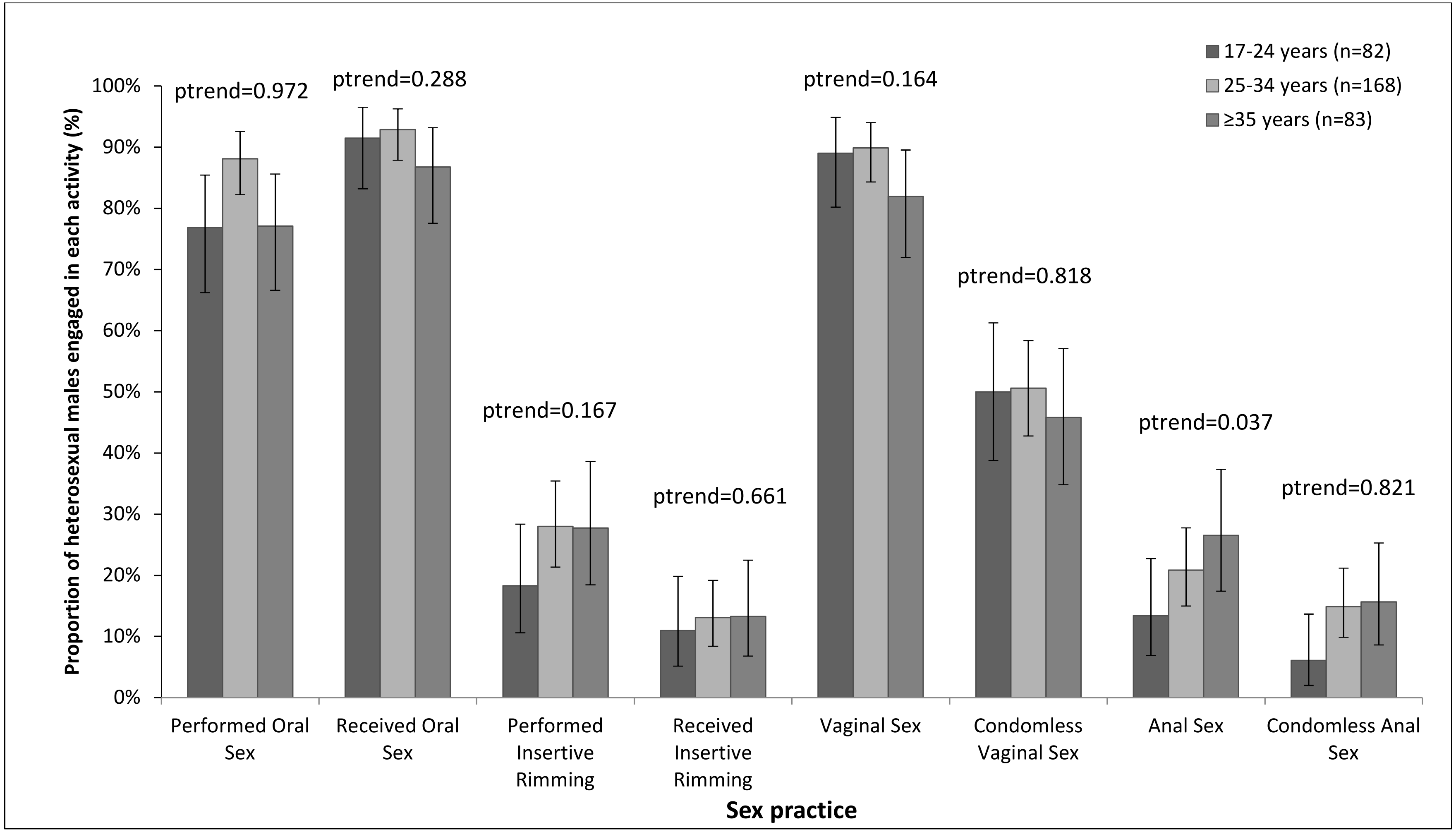

B.

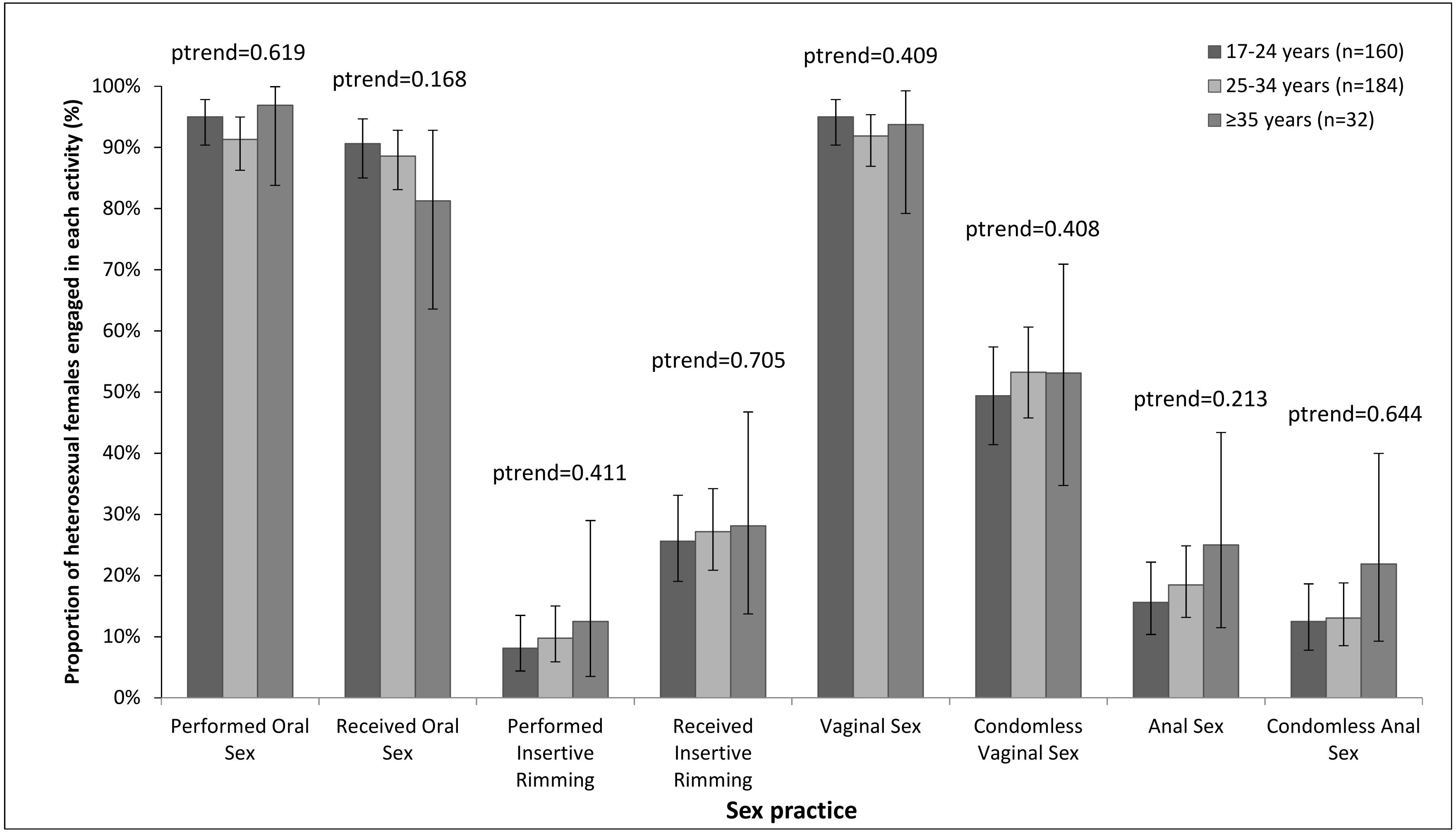

17-24 years (n=160)
25-34 years (n=184)
≥35 years (n=32)
Proportion of heterosexual females engaged in each activity (%)
100%
90%
80%
70%
60%
50%
40%
30%
20%
10%
0%
ptrend=0.619
ptrend=0.168
ptrend=0.411
ptrend=0.705
ptrend=0.409
ptrend=0.408
ptrend=0.213
ptrend=0.644
Performed Oral Sex
Received Oral Sex
Performed Insertive Rimming
Received Insertive Rimming
Vaginal Sex
Condomless Vaginal Sex
Anal Sex
Condomless Anal Sex
Sex practice

Supp. Table S1. Sexual practice of heterosexual men and women (n=709) in the previous three months.

	No. of individuals (%)	No. of men (%)	No. of women (%)	P-value*
Performed oral sex†				<0.001
No	83 (11.7)	58 (17.4)	25 (6.7)	
Yes	629 (88.3)	275 (82.6)	351 (93.4)	
Received oral sex				0.342
No	72 (10.2)	30 (9.0)	42 (11.2)	
Yes	637 (89.8)	303 (91.0)	334 (88.8)	
Performed insertive rimming‡				<.001
No	589 (83.1)	248 (74.5)	341 (90.7)	
Yes	120 (16.9)	85 (25.5)	35 (9.3)	
Received rimming**				<.001
No	567 (80.0)	291 (87.4)	276 (73.4)	
Yes	142 (20.0)	42 (12.6)	100 (26.6)	
Vaginal Sex				
No	66 (9.3)	41 (12.3)	25 (6.7)	0.010
Yes	643 (90.7)	292 (87.7)	351 (93.4)	
Condomless Vaginal sex				0.879
No	319 (47.1)	148 (47.4)	171 (46.9)	
Yes	358 (52.9)	164 (52.6)	194 (53.2)	
Anal Sex				0.379
No	574 (81.0)	265 (79.6)	309 (82.2)	
Yes	135 (19.0)	68 (20.4)	67 (17.8)	
Condomless Anal sex				0.072

No	54 (36.5)	33 (43.4)	21 (29.2)	
Yes	94 (63.5)	43 (56.6)	51 (70.8)	

* Using χ^2 test for significance

† Oral sex was defined as performing fellatio for females and performing cunnilingus for males

‡ Insertive rimming means participant's tongue in and around sex partner's anus

** Receiving rimming means partner's tongue in and around participant's anus

Supp. Table S2. Cross-tabulation of anal sex and insertive and receptive rimming among 709 heterosexual men (n=333) and women (n=376)

	**Insertive rimming**		**Received rimming**	
	**No**	**Yes**	**No**	**Yes**
Anal sex				
No	508	66	496	78
Yes	81	54	71	64
% Yes	13.8	45.0*	12.5	45.1*
95%CI	11.0-16.8	35.9-54.3	9.9-15.5	36.7-53.6
**Males**				
Anal sex				
No	216	49	248	17
Yes	32	36	43	25
%Yes	12.9	42.4*	14.8	59.5*
95%CI	9.0-17.7	31.7-53.5	10.9-19.4	43.2-74.4
**Females**				
Anal sex				
No	292	17	248	61
Yes	49	18	28	39
% Yes	14.4	51.4*	10.1	39.0*
95%CI	10.8-18.5	33.9-68.6	6.8-14.3	29.4-49.3

*Indicates significant difference, all p values were <0.001

Supp. Table S3. Sexual practice of heterosexual men and women (n=709) by age group in the previous three months.

	Aged 17-24 years (%)	Aged 25-34 years (%)	Aged ≥35 years (%)	Ptrend*
Performed oral sex[†]				0.181
No	27 (11.2)	36 (10.2)	20 (17.4)	
Yes	215 (88.8)	316 (89.8)	95 (82.6)	
Received oral sex				0.153
No	22 (9.1)	33 (9.4)	17 (14.8)	
Yes	220 (90.9)	319 (90.6)	98 (85.2)	
Performed insertive rimming[‡]				0.003
No	214 (88.4)	287 (81.5)	88 (76.5)	
Yes	28 (11.6)	65 (18.5)	27 (23.5)	
Received rimming**				0.533
No	192 (79.3)	280 (79.6)	95 (82.6)	
Yes	50 (20.7)	72 (20.4)	20 (17.4)	
Vaginal Sex				0.026
No	17 (7.0)	32 (9.1)	17 (14.8)	
Yes	225 (93.0)	320 (90.9)	98 (85.2)	
Condomless Vaginal sex				0.717
No	115 (48.9)	153 (45.5)	51 (48.1)	
Yes	120 (51.1)	183 (54.5)	55 (51.9)	
Anal Sex				0.011
No	206 (85.1)	282 (80.4)	85 (73.1)	
Yes	36 (14.9)	69 (19.6)	30 (26.1)	
Condomless Anal sex				0.891
No	14 (35.9)	28 (36.4)	12 (37.5)	

Yes	25 (64.1)	49 (63.6)	20 (62.5)	

* Using Cuzick's non-parametric test for trend for significance

† Oral sex was defined as performing fellatio for females and performing cunnilingus for males

‡ Insertive rimming means participant's tongue in and around sex partner's anus

** Receiving rimming means partner's tongue in and around participant's anus
